# Non-invasive visual theta entrainment modulates myelin-related and functional outcomes in an LPC-induced demyelination model

**DOI:** 10.1371/journal.pone.0357965

**Published:** 2026-09-15

**Authors:** Samaneh Dehghan, Kolsoum Dehdar, Nooshin Ahmadirad, Sarina Sadat Aboei Mehrizi, Mohammad Reza Raoufy, Mohammad Javan

**Affiliations:** 1 Eye Research Center, Moheb Kowsar Hospital, The Five Senses Institute, Iran University of Medical Sciences, Tehran, Iran; 2 Stem Cells and Regenerative Medicine Research Center, Iran University of Medical Sciences, Tehran, Iran; 3 JFK Neuroscience Institute, Hackensack Meridian Health JFK University Medical Center, Edison, New Jersey, United States of America; 4 Cellular and Molecular Research Center, Iran University of Medical Sciences, Tehran, Iran; 5 Department of Physiology, Faculty of Medical Sciences, Tarbiat Modares University, Tehran, Iran; 6 Institute for Brain Sciences and Cognition, Tarbiat Modares University, Tehran, Iran; 7 Department of Brain Sciences and Cognition, Cell Science Research Center, Royan Institute for Stem Cell Biology and Technology, ACECR, Tehran, Iran; Technion Israel Institute of Technology, ISRAEL

## Abstract

Demyelination is a hallmark of various neurodegenerative diseases, including multiple sclerosis (MS), leading to impaired neural transmission and motor deficits. The Lysophosphatidylcholine (LPC)-induced demyelination model is widely used because it reflects many aspects of demyelinating pathology. This study examined how non-invasive 4 Hz visual theta oscillation entrainment affects LPC-induced demyelination in the optic chiasm of male C57BL/6J mice. Mice received daily one-hour sessions of 4 Hz stimulation for two weeks post-LPC injection. The analysis of the VEPs showed that the latency of P1 waves was shorter in the theta stimulation group and significantly different from the LPC group on days 7 and 14. IBA1 immunostaining showed reduced IBA1 immunoreactivity at early post-lesion time points, while qPCR analysis showed lower *Aif-1* 1 and *Gfap* transcript levels in the theta-stimulated group. Luxol Fast Blue (LFB) and FluoroMyelin staining suggested greater preservation of myelin staining in the theta group at day 14. In parallel, molecular analyses revealed increased expression of oligodendrocyte-lineage markers, including *Pdfgra* at days 3 and 7 and *Olig2* and *Plp1* at day 14. Taken together, these findings suggest that theta oscillation entrainment modulates the molecular, cellular and electrophysiological responses that follow demyelinating injury, and that it may also influence the processes associated with myelin repair. Further studies are needed to clarify the mechanisms underlying these effects and to assess the therapeutic relevance of this non-invasive approach for demyelinating disorders.

## 1 Introduction

During myelination, progenitor cells of the oligodendrocyte lineage (OPCs) proliferate, differentiate into mature oligodendrocytes (OLs), and ensheathe axons in the white matter. In the adult central nervous system (CNS), demyelinating events can trigger a regenerative process known as remyelination, in which OPCs are recruited to the lesion site and differentiate to restore myelin sheaths around damaged axons. Efficient remyelination is essential for maintaining axonal integrity and preserving neuronal function [1–4]. However, remyelination often becomes incomplete or inefficient in chronic demyelinating conditions, resulting in progressive axonal degeneration and neurological impairment [2–4]. Although demyelinating diseases such as multiple sclerosis (MS) have highlighted the importance of remyelination in the CNS, many current therapeutic strategies mainly target inflammatory pathways and focus on limiting immune-mediated damage rather than directly promoting myelin repair [2,3,5–7]. Consequently, there remains a need for approaches that can support endogenous repair-related processes after demyelinating injury.

Remyelination is a complex and highly regulated process involving OPC proliferation, migration, differentiation, and maturation into myelinating oligodendrocytes. The success of this process is strongly influenced by the lesion microenvironment, including resident glial cells and local signaling mediators [8–10]. Among these cellular components, microglia contribute to the regenerative environment by participating in the clearance of inhibitory myelin debris and by releasing signals that can influence OPC recruitment and differentiation [11–13], Nevertheless, the capacity of OPCs to differentiate and generate mature oligodendrocytes declines under pathological conditions and during disease progression [14]. This differentiation block represents a major obstacle to successful remyelination and remains a key focus of regenerative neurobiology research. Indeed, several studies have shown that oligodendrocyte lineage cells are present within demyelinated lesions but often fail to differentiate efficiently into myelinating oligodendrocytes [15–17].

Recent evidence suggests that neuronal activity and network oscillations can influence glial biology and white matter structure. Activity-dependent mechanisms have been shown to regulate oligodendrocyte lineage progression and adaptive myelination in the CNS. Experimental studies indicate that externally induced brain oscillations can modulate glial responses and alter gene expression related to phagocytosis and cellular signaling pathways in microglia [11,18,19]. In particular, theta-frequency rhythms (3–8 Hz) have been associated with processes related to neural plasticity and white matter remodeling [20–23]. Previous work has demonstrated that theta activity can stimulate myelin production and enhance connectivity within specific neural circuits [24], suggesting that rhythmic neuronal activity may influence molecular pathways involved in oligodendrocyte maturation and myelin formation [25]. The ability to entrain brain rhythms through external stimulation therefore provides an opportunity to examine how specific oscillatory frequencies may affect glial and myelin-related responses without requiring complex behavioral training paradigms [26].

Experimental models of toxin-induced demyelination provide a useful platform for studying mechanisms of myelin loss and repair. Among these, the lysolecithin (LPC) model produces focal demyelinated lesions with a well-defined temporal sequence of demyelination followed by spontaneous remyelination [27]. This temporal profile allows investigation of cellular and molecular events associated with lesion development and recovery. In particular, demyelination of the optic chiasm provides a reproducible focal white matter injury model within the visual pathway, allowing structural and molecular analyses to be combined with functional assessment using visual evoked potentials.

Based on these considerations, we hypothesized that non-invasive visual theta oscillation entrainment (4 Hz) may modulate glial and oligodendrocyte-lineage-associated responses following LPC-induced demyelination in the optic chiasm. The objective of this study was to evaluate the effects of 4 Hz visual theta entrainment on functional, histological, immunofluorescence, and transcript-level outcomes during lesion development and recovery.

## 2 Materials and methods

### 2.1 Animals and experimental design

All experimental procedures were approved by the Institutional Ethics Committee of Iran University of Medical Sciences (IR.IUMS.REC.1401.425) and were conducted in accordance with international guidelines for the care and use of laboratory animals. A total of 35 adult male C57BL/6 mice were used in this study. Animals were assigned to three experimental groups: (1) intact control (n = 5), (2) LPC lesion (n = 15), and (3) LPC lesion with theta frequency visual entrainment (n = 15). Intact mice served as the physiological baseline and therefore did not undergo any intracranial injection. In contrast, LPC-injected mice without stimulation served as lesion controls for the stimulation group. To evaluate the temporal progression of demyelination and subsequent repair, animals in the LPC groups were examined at 3, 7, and 14 days after LPC injection, using independent cohorts at each time point (n = 5 per group per time point). Intact animals underwent longitudinal visual evoked potential (VEP) recordings at days 0, 3, 7, and 14 without lesion or stimulation. Animals in the stimulation group received 4 Hz visual flicker stimulation for one hour per day for fourteen consecutive days, beginning on the first day after the LPC injection. Following LPC injection, animals were randomly assigned to experimental groups, and outcome assessments were performed by investigators blinded to group allocation. The experimental unit was a single animal. The sample size (n = 5 per group per time point) was selected based on previous studies using similar experimental paradigms and practical considerations to balance biological replication with animal welfare. Each outcome measure was obtained from independent biological replicates, with each animal contributing a single independent observation to the statistical analysis.

Animals were housed under a 12 h light/12 h dark cycle with ad libitum access to food and water. To minimize circadian variability [28],theta visual flicker stimulation and VEP recordings were performed at approximately 13:00 each day. Tissue collection was carried out immediately after recordings at the same time of day for all experimental groups. Animal health and welfare were monitored daily throughout the study. Humane endpoint criteria included persistent distress, inability to access food or water, marked reduction in mobility, abnormal posture, or other signs of compromised health. No animals reached these criteria during the study, and euthanasia was performed only at predefined experimental endpoints for tissue collection. All surgical procedures were conducted under ketamine/xylazine anesthesia to minimize pain and distress. In intact animals, a single surgery was performed on day 0 for VEP electrode implantation. In the LPC and LPC+ theta groups, electrode implantation and LPC injection were performed during the same stereotaxic surgery to avoid a second anesthesia session. All procedures were carried out by experienced personnel, and no severe adverse effects were observed during the study.

### 2.2 Induction of demyelination

Focal demyelination was induced in the optic chiasm using LPC. The optic chiasm was demyelinated as described in our previous reports [29,30].

LPC-induced demyelination was performed under stereotaxic surgery. Each mouse was anesthetized with ketamine (70 mg/kg, i.p., Bremer Pharma, GmbH, Germany) and xylazine (10 mg/kg, i.p., Hoogstraten, Belgium) and LPC-induced demyelination was performed under stereotaxic surgery. Each mouse was anesthetized and placed in a stereotaxic frame, and the skull was exposed. A small burr hole was made at the midline coordinates, and a Hamilton syringe was lowered into the Optic chiasm. A total volume of 1 μL of 1% LPC (Sigma, St. Louis, USA) was injected at a rate of 0.2 μL/min into the optic chiasm using a Hamilton syringe (Hamilton Company, USA). Stereotaxic coordinates of the injection site were: anterior: +0.5 mm to Bregma, lateral: 0 mm, ventral: 4.9 mm from dura. After the injection, the needle was left in place for 5 minutes to prevent reflux and then slowly withdrawn. After surgery, animals were returned to their cages and allowed free access to food and water.

In the LPC and LPC+ Theta groups, this procedure was performed in the same surgical session as the electrode implantation. After completing both steps, mice were placed in a warmed cage and monitored until fully awake. Standard postoperative analgesia (meloxicam) was provided, and no antibiotics were required according to institutional guidelines for clean stereotaxic procedures.

### 2.3 Theta oscillation (4 Hz) visual flicker stimulation

Mice were acclimated daily for 30 minutes in a quiet environment prior to their placement in either the control or 4 Hz stimulation group. The 4 Hz stimulation using a general stimulator (BIODAC-A, TRITA Health Technology Co., Tehran, Iran) involved placing individual mice in a dark chamber illuminated by an LED bulb (12.5 ms on/off cycle, 60 W, 4 Hz) for one hour per day over two weeks in a soundproof room (McMaster-Carr, 5692T49). Local field potential (LFP) was recorded for confirming theta oscillation entrainment in the optic pathways and visual cortices. We applied 127 μm diameter electrodes (A.M. System Inc.) connected to a miniature buffer head stage having high-input impedance (BIODAC-A, TRITA Health Technology Co). The obtained signals were amplified with a 1000x gain, low-pass filtered <250 Hz, 50 Hz notch filter, and digitized at 1 kHz through a controller (BIODAC-ESR18622, TRITA Health Technology Co). Power spectral density (PSD) was computed using the Welch’s periodogram function in MATLAB.

### 2.4 Visual evoked potentials (VEPs)

Visual evoked potentials (VEPs) were recorded before and after demyelination to assess the functional integrity of the optic pathway, following our previously established protocol (Dehghan et al., 2021, 2016, 2012). Under anesthesia, electrodes were implanted and baseline VEPs were recorded during the same session. After induction of anesthesia, animals were positioned in a stereotaxic frame (Stoelting, USA). The recording electrode was placed over the primary visual cortex (AP −3.5 mm, ML +2.5 mm, DV −0.5 mm), and a reference electrode was positioned on the anterior skull. Electrodes were secured with dental cement, and animals were allowed to recover prior to subsequent procedures. A photo-stimulating LED was positioned 2 cm from the left eye, delivering light flashes contralateral to the VEP recording site. To eliminate light interference, the right eye was occluded and covered. A photo‑stimulating LED was positioned 2 cm from the left eye to deliver flashes contralateral to the recording site, while the right eye was covered to prevent light interference. Stimuli were administered 300 times at a frequency of 0.5 Hz using a general stimulator (BIODAC-A, TRITA Health Technology Co., Tehran, Iran). VEP waveforms were analyzed for latency and amplitude. The P1 latency was used as an index of demyelination, whereas the P1–N1 amplitude reflected axonal functional integrity.

In the intact control group, this was the only surgical procedure performed on day 0. In the LPC and LPC+theta groups, the electrode implantation was performed during the same surgery that included the LPC injection. For the LPC groups, VEPs were recorded at 3-, 7-, and 14-days post‑lesion. Independent cohorts were used for each time point (n = 5 per group per time point), and animals were euthanized immediately after recording for histological and molecular analysis. In contrast, the intact control group consisted of the same five mice, which underwent repeated VEP recordings on days 0, 3, 7, and 14. Tissue collection for this group was performed after the final VEP session on day 14. VEP signals were amplified, band‑pass filtered (1–100 Hz), digitized at 1 kHz, and averaged over 300 trials.

### 2.5 Myelin staining

Using myelin staining, we assessed the myelination status of the optic nerve across experimental groups (intact, LPC lesion, LPC lesion + theta stimulation).

After anesthesia, the animals were transcardially perfused and fixed. The optic nerves were cryoprotected and embedded in OCT compound (Bio-Optica), followed by serial sectioning with a cryostat microtome. Myelination was evaluated using Luxol Fast Blue (LFB) staining and Cresyl Fast Violet counterstaining. The remainder of the staining protocol followed our previously described method [31,32]. Briefly, mice were anesthetized with ketamine and xylazine and then subjected to transcardial perfusion with phosphate-buffered saline (PBS), followed by 4% paraformaldehyde (PFA). The tissues were subsequently fixed overnight in 4% PFA. After fixation, the tissues were cryoprotected in a sucrose solution, embedded in OCT compound (Bio Optica, Italy), and sectioned into 8 μm thick coronal slices using a cryostat microtome (Histo-line, Italy). These sections were stored at −80°C. For staining, the sections were rehydrated in PBS, immersed in 0.1% LFB (British Drug House, UK), and incubated at 60°C for 1 hour. Following staining, nuclei were counterstained with 0.1% Cresyl Violet (Merck, Germany), and the sections were then dehydrated and mounted using Entellan (Merck Chemicals, Germany).

FluoroMyelin (FM) staining was conducted to assess myelination in the optic nerves. Cryosections were first rehydrated with phosphate-buffered saline (PBS). The sections were then incubated with the FluoroMyelin staining solution, following the manufacturer’s instructions (1:300, f34652, ThermoFisher, Oregon, USA). Quantification of demyelinated areas was performed using a plugin for ImageJ.

### 2.6 Immunohistofluorescence analysis

To evaluate the effect of visual oscillation stimuli on myelin repair and the presence of oligodendrocyte precursor cells (OPCs), microglia, and mature oligodendrocytes in various experimental groups, optic chiasm sections were immunostained. To increase membrane permeability, sections of 8 µm were washed three times in phosphate-buffered saline (PBS) followed by 15 minutes of treatment with 0.1% Triton X-100. After blocking with appropriate normal serum, primary antibodies were applied: myelin basic protein (MBP, Aveslabs, 1:500), OLIG2(Abcam, Inc. ab9610, 1:200), PDGFRα (Santa Cruz Biotechnology, 1:100), GFAP (Dako, 1:500) and IBA1(FUJIFILM Wako Pure Chemical, 1: 500). Following PBS washes, sections were incubated with secondary antibodies (goat anti-rabbit IgG; A-11008, donkey anti-goat IgGA11036, and rabbit anti-chicken; ab6751, each at 1:1000 dilution) for one hour at room temperature. Nuclei were stained with DAPI (Santa Cruz Biotechnology Inc., CA, sc-24941), and sections were mounted using Ultracruz mounting medium (Santa Cruz Biotechnology, SC 24941, USA). Images were acquired with a BX-51 fluorescent microscope (Olympus Optical Co., Ltd., Tokyo, Japan).

Five animals per group at each time point were evaluated for histological and immunofluorescence analyses. For each animal, three slides were analyzed, with three coronal sections containing the entire optic chiasm examined per slide (nine sections per animal). Two adjacent images were acquired per section and merged using Adobe Photoshop Auto-Photomerge. The entire optic chiasm was defined as the ROI, and image acquisition and analysis were performed under blinded conditions. Measurements from multiple sections were averaged to obtain one representative value per animal for statistical analysis.

### 2.7 Gene expression

To further investigate the number of oligodendrocyte lineage cells and confirm myelination, real-time PCR was conducted targeting various markers of oligodendrocyte lineage cells. Additionally, the expression of phagocytosis-related genes in microglia was assessed to provide further insights into the role of microglia in the remyelination process.

For gene expression analysis, optic chiasm tissue containing the LPC lesion site was micro-dissected from each animal at the predefined experimental endpoints. Total RNA was isolated from these micro-dissected optic chiasm samples using the High Pure RNA Tissue Kit (Roche, Germany; Catalog No. 12033674001), according to the manufacturer’s protocol. The RNA was reverse-transcribed into cDNA using cDNA synthesis kit (Parstous, biotechnology, Iran). Quantitative real-time PCR (q-PCR) was conducted using the Real Q-PCR Master Mix Kit (Ampliqon, Herlev, Denmark; Catalog No. 250507) on a Rotor-Gene Q device (Qiagen, Hilden, Germany). The q-PCR conditions were set as follows: an initial denaturation step at 95 °C for 15 minutes, followed by 35 amplification cycles with each cycle consisting of 60 seconds at 95 °C, 60 seconds at the specified annealing temperature, and 60 seconds at 72 °C. Annealing temperatures were 63 °C for *Gapdh, 60°C* fo*r Aif-1, Gfap, Pdgfra, Olig2,* and *Plp1*. All reactions were performed in duplicate. GAPDH served as the endogenous control to adjust for sample variation, and the relative expression of target genes was determined using the delta-delta-Ct method. Primer sequences are detailed in Table 1.

**Table 1:** Primer sequences.

Gene and Accession number	Primer sequences
*Aif-*1 NM_019467.4	F: TGAGGAGCCATGAGCCAAAG R: GCTTCAAGTTTGGACGCCAG
*Gfap* NM_001131020.1	F: TCTAAGGGAGAGCTGGCAGGGCT R: AGGGCGAAGAAAACCGCATCACC
*Pdgfra* NM_001347719.1	F: GGTGGCTCCCACTCTGCGATC R: CGTAAGGCAACTGCATGGGGTC
*Olig2* NM_016967.2	F: CCAGCGGAACCCCGAAAGGT R: ACCAGGCTGGCGTCCGAGT
*Plp1* NM_011123.4	F: GCCAAAGACATGGGCTTGTT R: GGTGGTCTTGTAGTCGCCAA

### 2.8 Hematoxylin and eosin (H&E) staining

Hematoxylin and Eosin (H&E) staining was conducted to evaluate microglia infiltration at day 3 post LPC injection. For staining, the cryosections were rehydrated with phosphate-buffered saline (PBS), then stained with Hematoxylin for 4 minutes. After staining, the sections were cleared in xylene, counterstained with Eosin for 1.5 minutes, and then washed and coverslipped. Microglia infiltration was assessed by examining the stained sections under an Olympus BX51 microscope, and images were captured using a DP-72 camera (BX51 TRF, USA).

### 2.9 Statistical analysis

Statistical analyses were performed using GraphPad Prism. Data normality was verified with the Shapiro-Wilk test. Since animals at each time point were from independent cohorts, treatment and time were analyzed as between‑subject factors using a two‑way ANOVA, followed by Tukey’s post hoc tests when applicable. For datasets measured at a single time point, such as Aif1 transcript levels in Fig 4A, group differences were analyzed using ordinary one-way ANOVA followed by Tukey’s multiple-comparisons test. Exact F-values, degrees of freedom, and p-values are reported in the Results. Statistical significance was set at p < 0.05, and no animals or data points were excluded. The unit of analysis for all datasets was a single animal. The sample sizes were determined based on the specific experimental endpoint and are indicated in the corresponding analyses and figure legends. The animal was designated as the experimental unit for all analyses.

**Fig 1:**
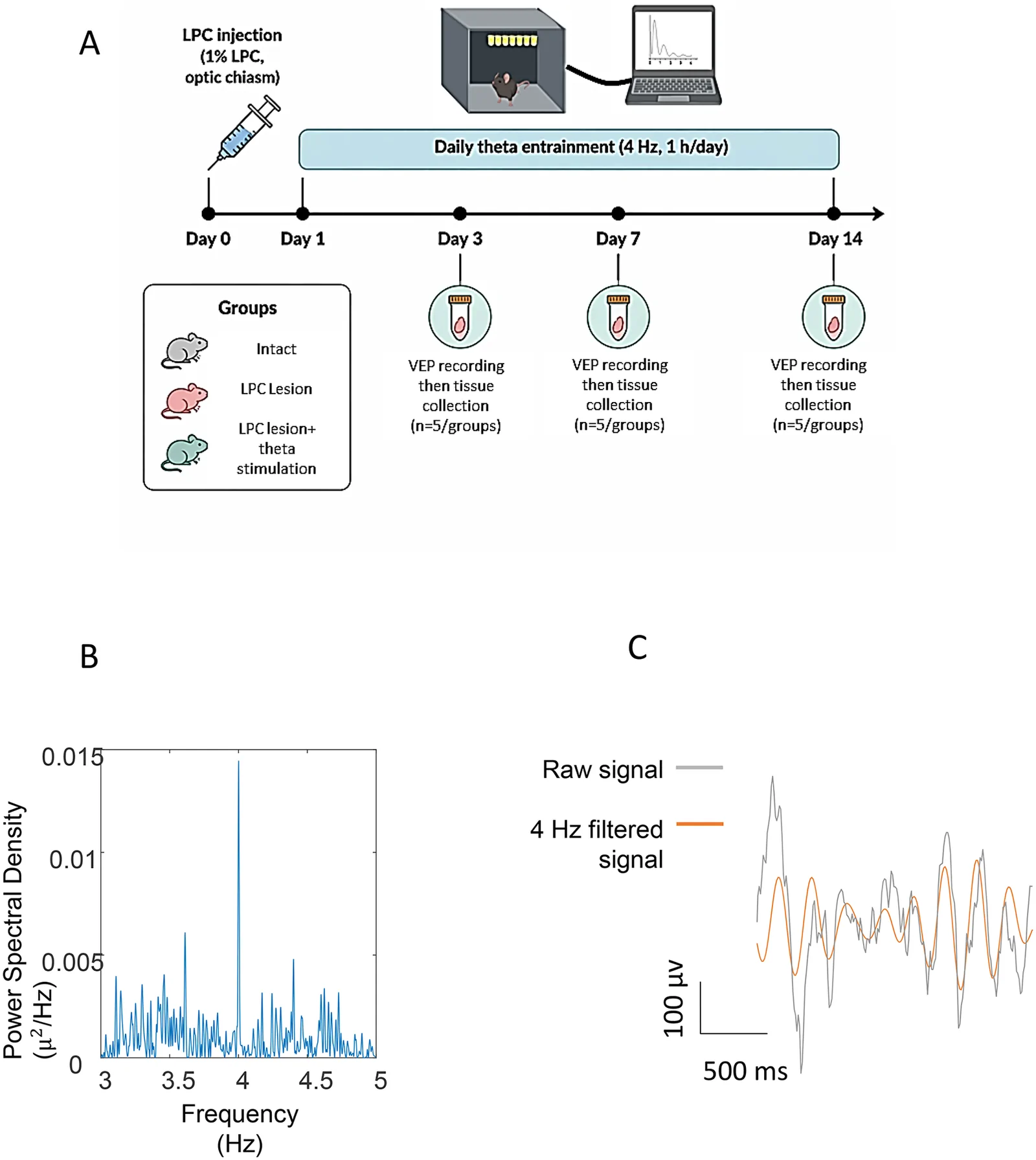
Experimental timeline and confirmation of 4 Hz visual entrainment. (A)Schematic experimental timeline. Focal demyelination was induced by 1% LPC injection into the optic chiasm on day 0. Animals in the stimulation group received daily theta-frequency visual entrainment (4 Hz, 1 h/day) from day 1 to day 14. Independent cohorts of LPC and LPC+Theta animals were euthanized at 3-, 7-, and 14-days post-lesion for tissue collection (n = 5 per group per time point). Outcome measures included VEP recording, myelin staining, oligodendrocyte-lineage marker analysis, and glial-associated marker analysis. (B, C) Visual theta stimulation increased 4 Hz oscillatory power in the visual cortex.

**Fig 2:**
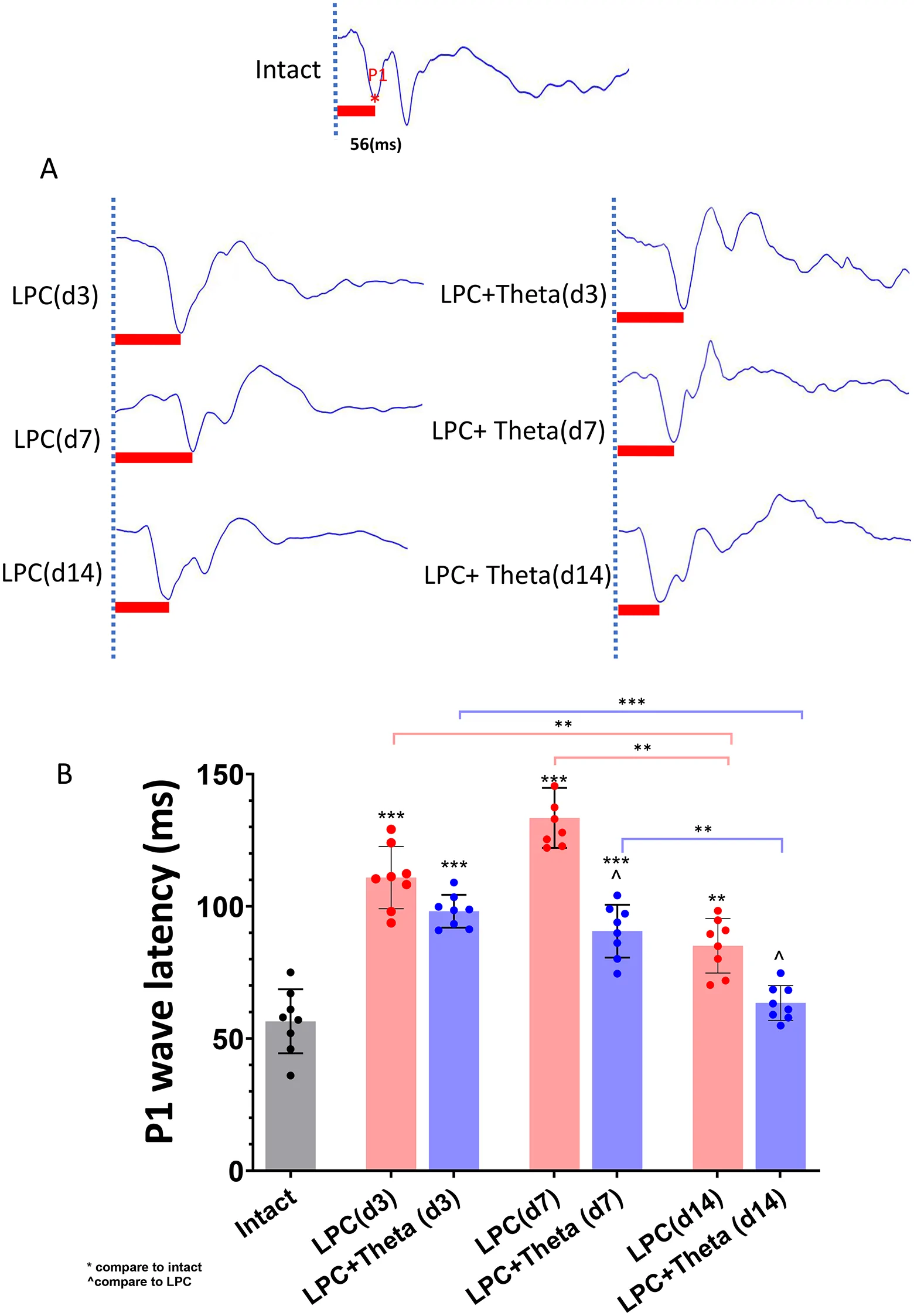
Theta entrainment reduces P1 latency after LPC-induced demyelination. (A) Representative VEP recordings from experimental groups at days 3, 7, and 14 post-lesion. (B) Quantification of P1 wave latency across the Intact, LPC, and LPC+Theta groups at days 3, 7, and 14. Data are presented as mean ± SEM. Statistical analysis was performed using two-way ANOVA followed by Tukey’s multiple-comparisons test. *p < 0.05, **p < 0.01, ***p < 0.001 versus Intact; ^p < 0.05, ^^p < 0.01, ^^^p < 0.001 versus the corresponding LPC group.

**Fig 3:**
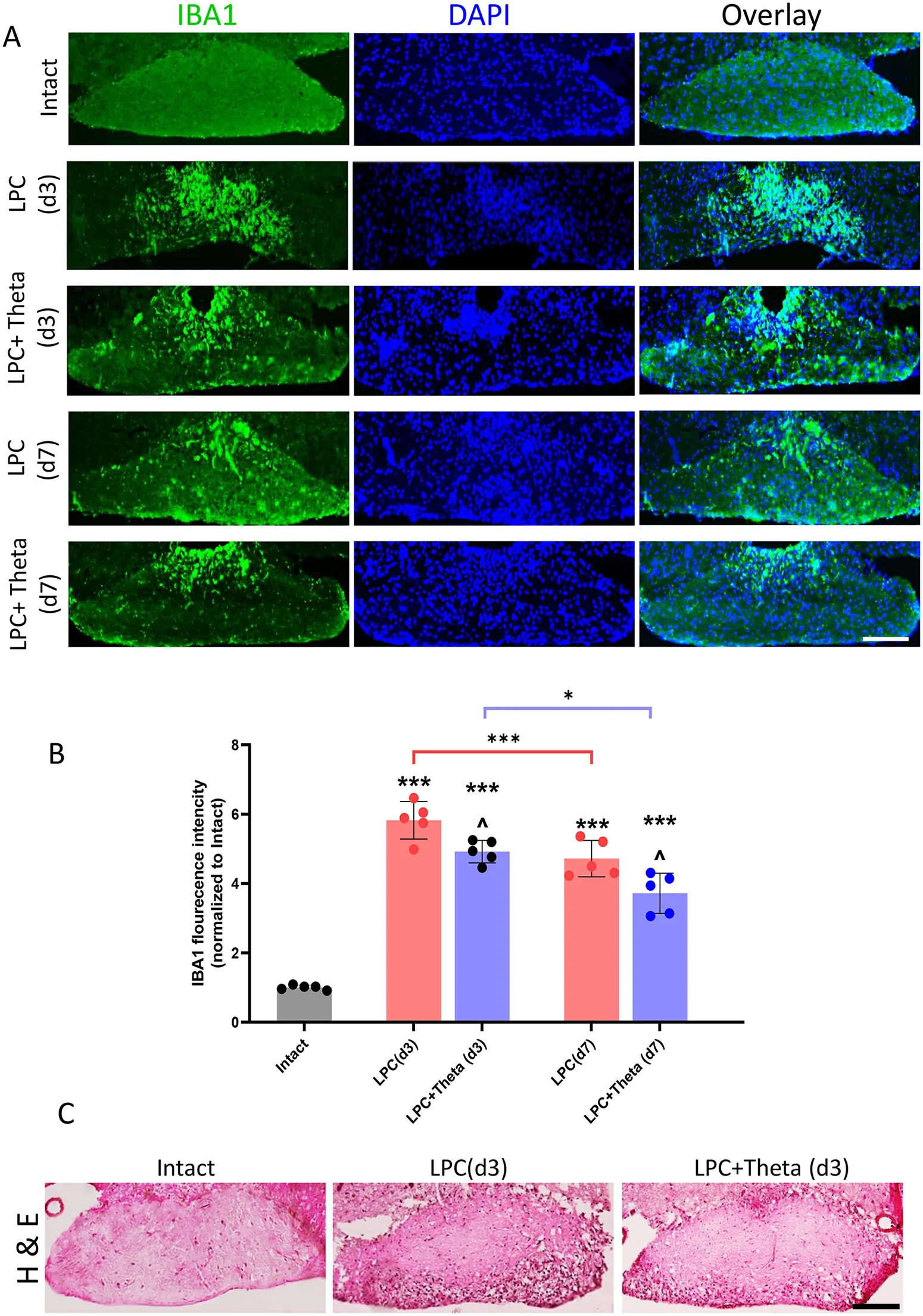
Theta entrainment alters IBA1 immunoreactivity and tissue morphology after LPC-induced demyelination. (A)Representative immunofluorescence images showing IBA1 immunoreactivity (green) and DAPI nuclear staining (blue) in the Intact, LPC day 3, LPC day 7, LPC+Theta day 3, and LPC+Theta day 7 groups. (B) Quantification of IBA1 fluorescence intensity normalized to the Intact group. Data are presented as mean ± SEM. Statistical analysis was performed using two-way ANOVA followed by Tukey’s multiple-comparisons test. ***p < 0.001 versus Intact; ^p < 0.05 versus the corresponding LPC group. (C) Representative hematoxylin and eosin staining of coronal optic chiasm sections at day 3. Dashed lines indicate the lesion/demyelinated area.

## 3 Results

We examined the effects of visual stimulus on the optic chiasm in order to determine whether visual stimulus influences the demyelination process caused by LPC. The optic chiasm of the animals after LPC injection showed signs of demyelination two days after injection. Here, LPC was injected to optic chiasm of adult male C57BL/6J mice. A theta oscillation entrainment (4 Hz) was applied 1 h/d for 2 weeks to mice at that point (Fig 1A).

To investigate whether 4 Hz visual stimulus can entrain theta oscillations in optic pathways, and visual cortices of mice, we conducted recordings of LFP during visual stimulation at 4 Hz (Fig 1A). In response to visual stimulation, LFP power was clearly elevated at the 4 Hz frequency band (Fig 1B, C).

### 3.1 Theta oscillation entrainment is associated with reduced P1 latency following LPC-induced demyelination

P1 wave latency in VEP recordings was analyzed to assess changes in visual pathway conduction following LPC induced demyelination and theta stimulation on days 3, 7, and 14 (Fig 2A). In the intact group, conduction time in the visual pathway was approximately 56 milliseconds. P1 wave latency increased after LPC induced demyelination across all examined time points. There was substantial P1 latency prolongation in the LPC(d3) group compared with the intact group (***p < 0.001). A similar increase in latency was observed in the LPC(d7) group (***p < 0.001 vs. intact). By day 14, the LPC(d14) group showed a reduction in latency (**p < 0.01), but latency remained significantly prolonged compared with the intact group. Theta oscillation entrainment was associated with reduced P1 latency compared with LPC alone. In the LPC+Theta(d3) group, latency was lower than in LPC(d3), although this difference was not statistically significant. At day 7, latency was reduced in the LPC+Theta (d7) group compared with LPC(d7) (^p < 0.05), while remaining elevated relative to the intact group (***P < 0.001). On day 14, the LPC+Theta(d14) group exhibited a further reduction in latency (^p < 0.05 vs. LPC(d14)), and was not significantly different from the intact group (Fig 2B). Two-way ANOVA revealed significant effects of experimental group (F(2,63)=17.20, p < 0.0001), time (F(2,63)=75.56, p < 0.0001), and the interaction between group and time (F(4,63)=4.686, p = 0.0022) on P1 wave latency. Together, these findings indicate that theta entrainment was associated with partial normalization of visual pathway conduction after LPC-induced demyelination.

### 3.2 Theta entrainment reduces IBA1 immunoreactivity following LPC-induced demyelination immunohistochemical analysis

At days 3 and 7, immunohistochemical staining for IBA1 and DAPI was performed to evaluate changes in IBA1 immunoreactivity following LPC‑induced demyelination and the potential modulatory effect of theta oscillation entrainment (Fig 3A, B). In the intact group, IBA1 fluorescence intensity was low, indicating minimal basal expression. LPC injection produced a marked increase in IBA1 staining, with significantly elevated fluorescence intensity observed at day 3 compared with the intact group (***p < 0.001). Elevated IBA1 protein expression persisted at day 7 in the LPC group (***p < 0.001 vs. intact). Theta oscillation entrainment was associated with reduced IBA1 fluorescence intensity. In the LPC+Theta Day 3 group, IBA1 levels were significantly lower than in the LPC day 3 group (^p < 0.05), although still higher than in intact tissue (***p < 0.001). A similar reduction was observed at day 7, where IBA1 fluorescence intensity in the LPC+Theta group was significantly decreased compared with the LPC day 7 group (^p < 0.05). Two‑way ANOVA revealed a significant main effect of experimental group (intact, LPC, and LPC+Theta; F (2,24) =41.96, p < 0.0001) and time (days 3 and 7; F (2,24) =10.68, p = 0.0005), while the interaction between group and time did not reach statistical significance (F (4,24) =2.705, p = 0.0544). H&E staining was used to evaluate optic nerve morphology and tissue integrity. The intact optic nerve showed a well‑preserved structure with no inflammatory cell infiltration. In the LPC (day 3) group, marked tissue disorganization was observed, accompanied by loss of normal architecture and infiltration of inflammatory cells. In contrast, the LPC+Theta (day 3) group showed better-preserved tissue organization and less apparent inflammatory infiltration compared with the LPC group (Fig 3C). These findings indicate that theta entrainment was associated with reduced IBA1 immunoreactivity during the early post-lesion period.

### 3.3 Theta oscillation reduces LPC‑induced *Aif-1* and *Gfap* gene expression

**Fig 4:**
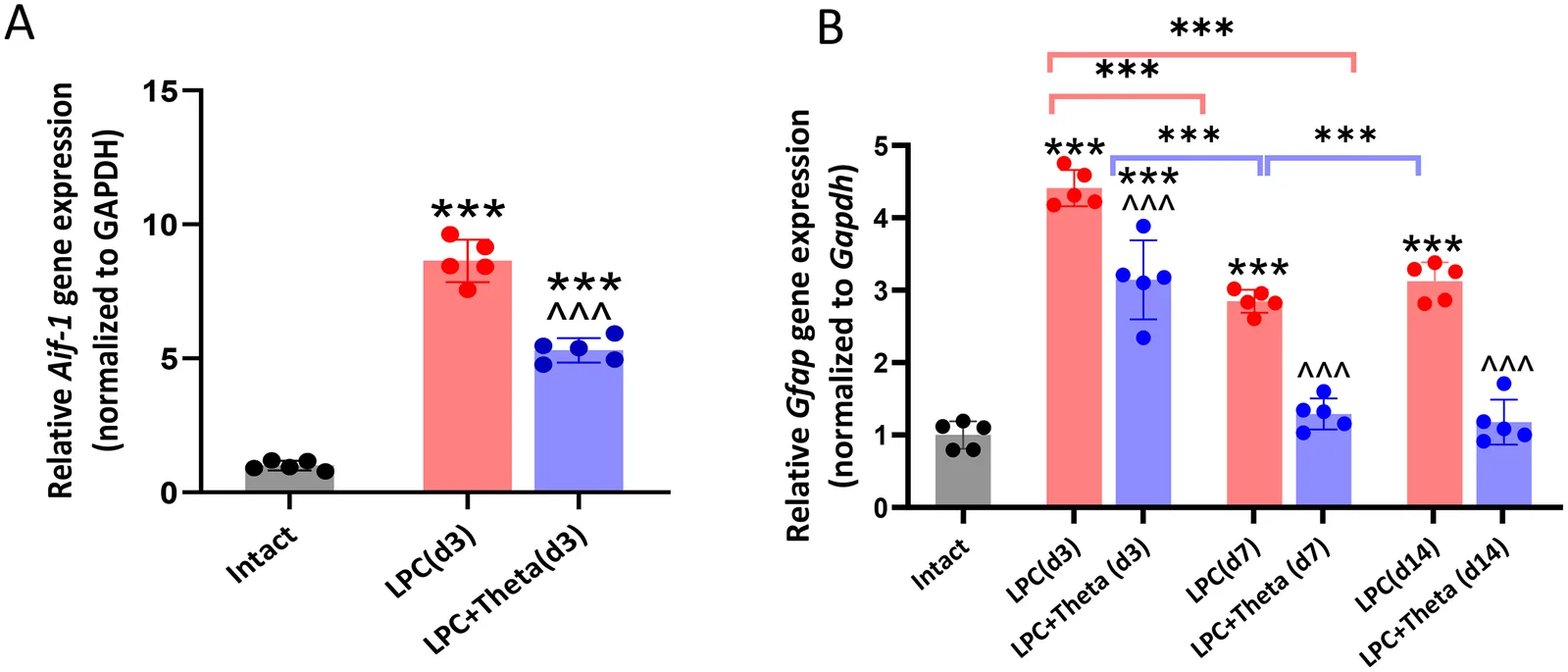
*Aif-1* and *Gfap* transcript levels after LPC-induced demyelination. (A) *Aif-1* transcript levels encoding *Aif-1*, normalized to *Gapdh*, in the Intact, LPC day 3, and LPC+Theta day 3 groups. Data were analyzed using ordinary one-way ANOVA followed by Tukey’s multiple comparisons test because this dataset included a single time point. (B) *Gfap* transcript levels encoding GFAP, normalized to *Gapdh*, across experimental groups at days 3, 7, and 14. Data were analyzed using two-way ANOVA followed by Tukey’s multiple-comparisons test, with experimental group and time as between-subject factors. Data are presented as mean ± SEM. *p < 0.05, **p < 0.01, ***p < 0.001 versus Intact; ^p < 0.05, ^^p < 0.01, ^^^p < 0.001 versus the corresponding LPC group.

The expression levels of *Aif-1* and *Gfap* genes were evaluated following LPC injection and theta oscillation entrainment (Fig 4). Relative *Aif-1* transcript levels, encoding the microglial marker AIF-1, were increased after LPC-induced demyelination in the LPC(d3) group compared with the intact group (***p < 0.001) (Fig 4A). Theta oscillation entrainment significantly reduced *Aif-1* expression in the LPC+Theta(d3) group compared with LPC alone (***p < 0.001), although expression levels remained elevated relative to the intact group (***p < 0.001).

*Gfap transcript levels encoding GFAP* (Fig 4B), a commonly used astrocyte‑associated marker, was also significantly increased following LPC injection. At day 3, *Gfap* expression in the LPC group was significantly higher than in the intact group (***p < 0.001). Theta oscillation entrainment significantly reduced *Gfap* gene expression in the LPC+Theta(d3) group compared with LPC (^^^p < 0.001). *Gfap* expression in the LPC group decreased gradually at days 7 and 14, while lower expression levels were observed in the LPC+Theta groups (***p < 0.001). Two‑way ANOVA demonstrated significant effects of experimental group (intact, LPC, and LPC+Theta; F (2,46)=113.3, p < 0.0001), time (days 3, 7, and 14; F(2,46)=167.5, p < 0.0001), and their interaction (F(4,46)=39.16, p < 0.0001) on *Gfap* gene expression. These results suggest that theta entrainment modulated microglial and astrocyte associated transcript responses after LPC-induced demyelination.

### 3.4 LPC induced demyelination in the optic chiasm and the effect of theta oscillation entrainment

**Fig 5:**
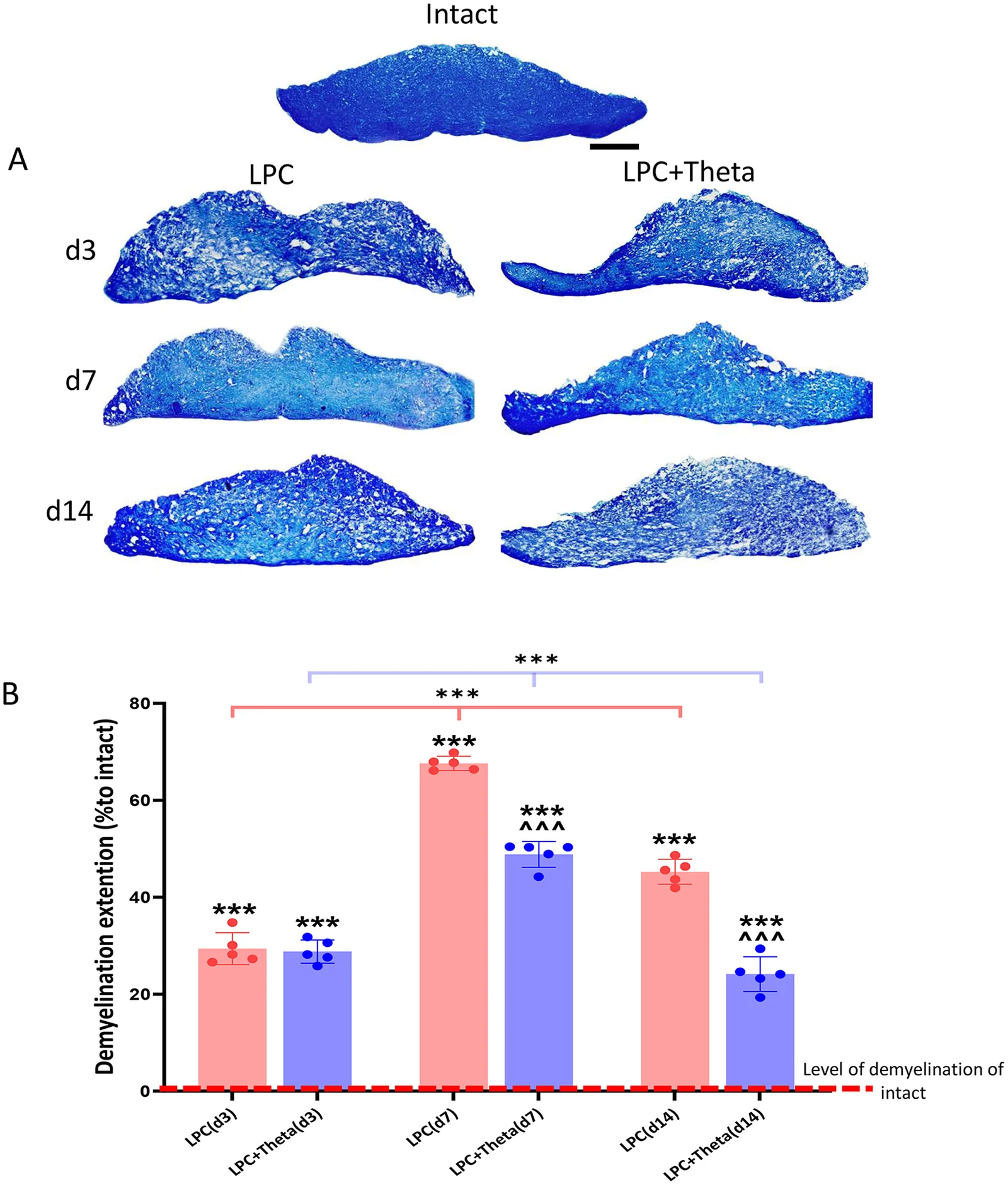
Theta entrainment is associated with reduced myelin loss after LPC-induced demyelination in the optic chiasm. (A) Representative Luxol Fast Blue (LFB)-stained optic chiasm sections from the Intact, LPC, and LPC+Theta groups at days 3, 7, and 14 post-lesion. (B) Quantification of the demyelinated area expressed as a percentage relative to the analyzed optic chiasm area. Data are presented as mean ± SEM. Statistical analysis was performed using two-way ANOVA followed by Tukey’s multiple-comparisons test. ***p < 0.001 versus Intact; ^^^p < 0.001 versus the corresponding LPC group.

After LPC injection, optic chiasm sections were stained with Luxol Fast Blue (LFB) at days 3, 7, and 14 to assess the extent of demyelination and the impact of theta oscillation entrainment (Fig 5A). In the optic chiasm, LPC caused significant demyelination, as indicated by reduced LFB staining intensity. At all examined time points, myelin density was significantly lower than in the intact group. On day 3 post LPC injection, the optic chiasm showed moderate demyelination (***p < 0.001 vs. intact). Compared with the intact group, the demyelinated area reached its maximum at day 7 (***p < 0.001). By day 14, a reduction in demyelinated area was observed relative to day 7, but demyelination remained significantly elevated compared with intact levels (***p < 0.001). Theta oscillation entrainment following LPC induced injury was associated with reduced demyelination at days 7 and 14 compared with LPC alone. On day 3, there was no significant difference between the LPC and LPC+Theta groups. At day 7, when demyelination peaked in the LPC group, the LPC+Theta(d7) group exhibited a marked reduction in demyelination (^^^p < 0.001 vs. LPC(d7)). By day 14, this reduction persisted, with the demyelinated area in the LPC+Theta(d14) group (~25%) significantly lower than in the LPC(d14) group (~40%) (^^^p < 0.001) (Fig 5B). However, demyelination remained significantly higher than in the intact group at all time points. Two-way ANOVA demonstrated significant effects of experimental group (intact, LPC, and LPC+Theta; F(2,36)=1778, p < 0.0001), time (days 3, 7, and 14; F(2,36)=316.2, p < 0.0001), and their interaction (F(4,36)=110.3, p < 0.0001) on the extent of demyelination. These findings indicate that theta entrainment was associated with reduced myelin loss after LPC lesion; however, these data do not distinguish myelin preservation from accelerated remyelination.

### 3.5 Theta oscillation entrainment modulates OPC‑related gene expression during demyelination and recovery

**Fig 6:**
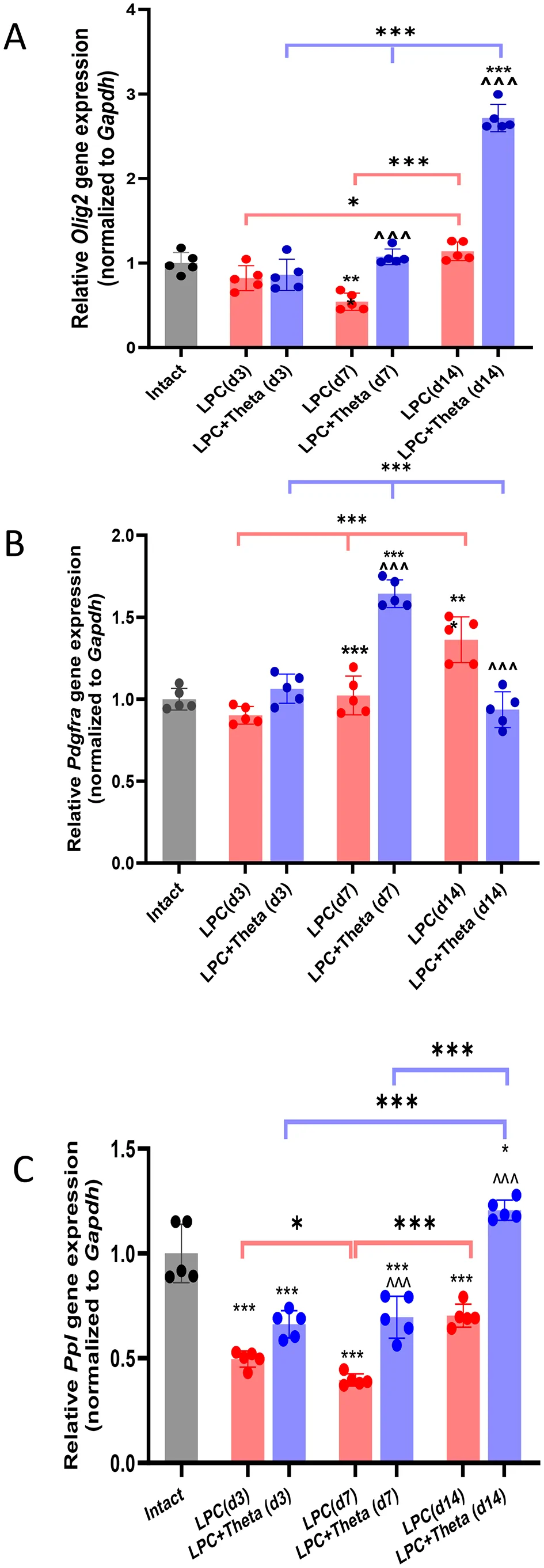
Oligodendrocyte-lineage-associated transcript levels after LPC-induced demyelination. (A) *Olig2* transcript levels normalized to *Gapdh* across experimental groups at days 3, 7, and 14. (B) *Pdgfra* transcript levels normalized to *Gapdh* across experimental groups at days 3, 7, and 14. (C) *Plp1* transcript levels normalized to *Gapdh* across experimental groups at days 3, 7, and 14. Data are presented as mean ± SEM. Statistical analysis was performed using two-way ANOVA followed by Tukey’s multiple-comparisons test. *p < 0.05, **p < 0.01, ***p < 0.001 versus Intact; ^p < 0.05, ^^p < 0.01, ^^^p < 0.001 versus the corresponding LPC group.

*Olig2* transcript levels, as an oligodendrocyte lineage associated marker, were altered following LPC-induced demyelination (Fig 6A). In the LPC group, *Olig2* transcript levels were significantly decreased at day 7 compared with the intact group (***p < 0.001), consistent with oligodendrocyte-lineage disruption during the peak demyelination phase. Theta oscillation entrainment was associated with higher *Olig2* transcript levels compared with the corresponding LPC group, with significant differences observed at day 3 (^^p < 0.01) and day 7 (^^^p < 0.001). At day 14, *Olig2* transcript levels remained higher in the LPC+Theta group than in the LPC group (^^p < 0.01). Two-way ANOVA revealed significant effects of experimental group (intact, LPC, and LPC+Theta; F(2,36)=118.5, p < 0.0001), time (days 3, 7, and 14; F(2,36)=152.2, p < 0.0001), and the group × time interaction (F(4,36)=81.40, p < 0.0001) on *Olig2* transcript levels. These findings indicate that theta entrainment modulated oligodendrocyte-lineage-associated transcript responses after LPC-induced demyelination; however, *Olig2* expression alone should not be interpreted as direct evidence of accelerated OPC differentiation.

*Pdgfra* transcript levels encoding PDGFRα, a well-established marker of oligodendrocyte precursor cells (OPCs), increased expression following LPC-induced demyelination (Fig 6B). Expression was significantly elevated at day 7 compared with the intact group and day 3 (***p < 0.001). Theta stimulation was associated with higher *Pdgfra* expression at both day 3 (^^p < 0.01) and day 7 (^^^p < 0.001) compared with the LPC group. By day 14, *Pdgfra* levels declined in both groups, although expression remained higher in the LPC+Theta group (^p < 0.05). Two‑way ANOVA demonstrated significant effects of experimental group (intact, LPC, and LPC+Theta; F (2,36) =49.08, p < 0.0001), time (days 3, 7, and 14; F(2,36)=52.82, p < 0.0001), and their interaction (F(4,36)=142.6, p < 0.0001) on *Pdgfra* expression.

*Plp1 transcript levels encoding* PLP, a myelin-associated marker, was markedly reduced in the LPC group at all examined time points compared with the intact group (***p < 0.001) (Fig 6C). Although *Plp1* levels remained low following LPC-induced demyelination, theta oscillation entrainment was associated with a significant increase in *Plp1* expression at day 14 compared with the LPC group (^^^p < 0.001). Despite this increase, *Plp1* expression in the LPC+Theta group remained significantly lower than in intact tissue (***p < 0.001). Two-way ANOVA demonstrated significant effects of experimental group (intact, LPC, and LPC+Theta; F(2,36)=97.06, p < 0.0001), time (days 3, 7, and 14; F(2,36)=38.60, p < 0.0001), and their interaction (F(4,36)=13.71, p < 0.0001) on *Plp1* expression. These findings indicate that theta entrainment modulated oligodendrocyte-lineage-associated transcript responses after LPC-induced demyelination; however, these marker changes should not be interpreted as direct evidence of accelerated OPC differentiation.

### 3.6 Fluoromyelin staining reveals the effect of theta treatment on LPC-induced myelin loss in the optic chiasm

**Fig 7:**
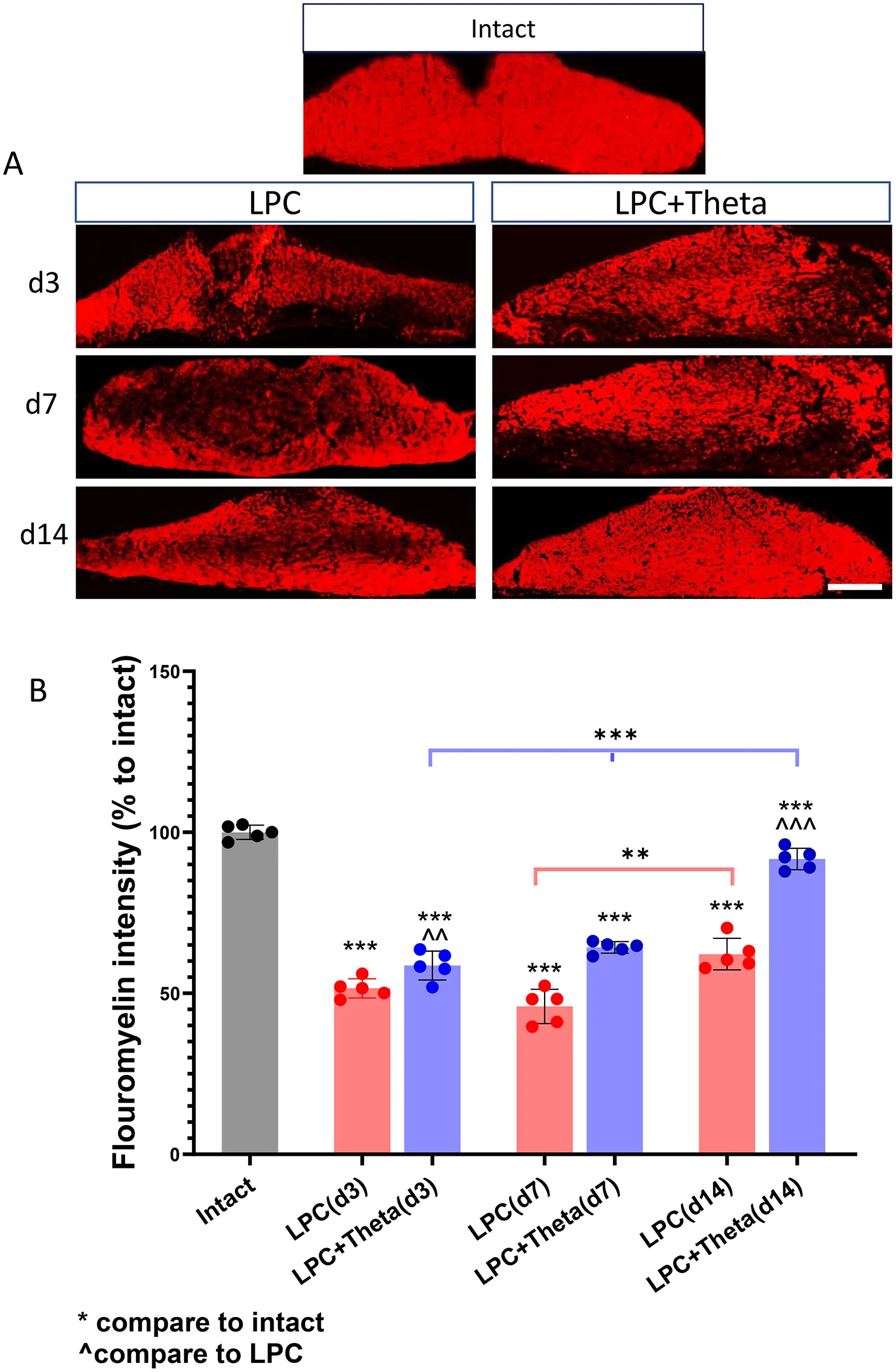
FluoroMyelin staining shows changes in myelin content after LPC-induced demyelination. (A) Representative FluoroMyelin-stained optic chiasm sections from the Intact, LPC, and LPC+Theta groups at days 3, 7, and 14 post-lesion. (B) Quantification of FluoroMyelin fluorescence intensity normalized to the Intact group. Data are presented as mean ± SEM. Statistical analysis was performed using two-way ANOVA followed by Tukey’s multiple-comparisons test. ***p < 0.001 versus Intact; ^p < 0.05, ^^p < 0.01, ^^^p < 0.001 versus the corresponding LPC group.

Fluoromyelin staining was performed on optic chiasm sections at 3, 7, and 14 days post LPC injection to evaluate myelin content following LPC injection and theta oscillation entrainment. Fluoromyelin intensity was quantified as an indicator of myelin content and compared with the intact group (Fig 7A). LPC significantly reduced fluoromyelin intensity compared with intact at all examined time points, indicating substantial myelin loss in the optic chiasm. At day 3, the LPC group showed a marked reduction in fluoromyelin intensity (***p < 0.001 vs. intact). Myelin loss increased further by day 7 (***p < 0.001 vs. intact), representing the peak of demyelination. By day 14, fluoromyelin intensity increased relative to days 3 and 7 (**p < 0.01 vs. day 3 and day 7), although it remained significantly lower than the intact group (***p < 0.001 vs. intact). Higher fluoromyelin intensity was observed in the LPC+Theta groups compared with LPC alone. At day 3, the LPC+Theta group remained significantly lower than the intact group (***p < 0.01 vs. intact) but showed higher staining than LPC(d3) (^^p < 0.01 vs. LPC). At day 7, the LPC+Theta group exhibited greater fluoromyelin staining than LPC(d7), although this difference was not statistically significant and values remained lower than intact. By day 14, fluoromyelin intensity increased further in the LPC+Theta group and was not significantly different from intact, while remaining significantly higher than LPC(d14) (^^^p < 0.001) (Fig 7B). Two-way ANOVA demonstrated significant effects of experimental group (intact, LPC, and LPC+Theta; F (2,36)=343.1, p < 0.0001), time (days 3, 7, and 14; F(2,36)=48.97, p < 0.0001), and their interaction (F(4,36)=19.78, p < 0.0001) on FluoroMyelin intensity. Because differences between the LPC and LPC+Theta groups were already detectable at earlier time points, the present design does not allow a clear distinction between reduced initial demyelination and accelerated remyelination. These findings further support an association between theta entrainment and higher myelin-related staining intensity during the lesion-recovery period.

### 3.7 Theta oscillation entrainment modulates PDGFRα, OLIG2, and MBP protein expression during remyelination

**Fig 8:**
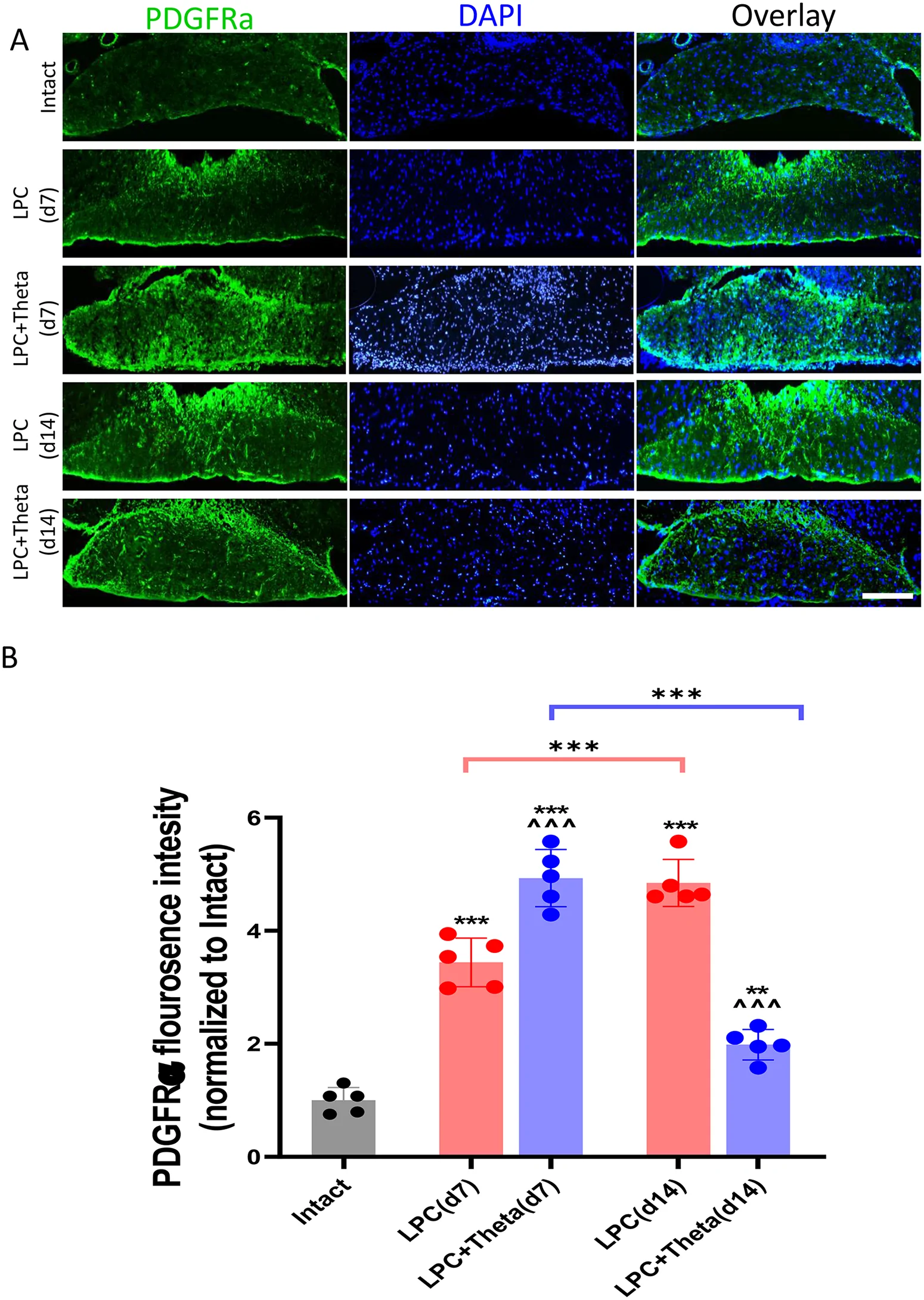
PDGFRα immunoreactivity after LPC-induced demyelination and theta entrainment. (A) Representative immunofluorescence images showing PDGFRα immunoreactivity (red) and DAPI nuclear staining (blue) in the Intact, LPC day 7, LPC+Theta day 7, LPC day 14, and LPC+Theta day 14 groups. (B) Quantification of PDGFRα fluorescence intensity normalized to the Intact group. Data are presented as mean ± SEM. Statistical analysis was performed using two-way ANOVA followed by Tukey’s multiple-comparisons test. ***p < 0.001 versus Intact; ^^p < 0.01, ^^^p < 0.001 versus the corresponding LPC group.

**Fig 9:**
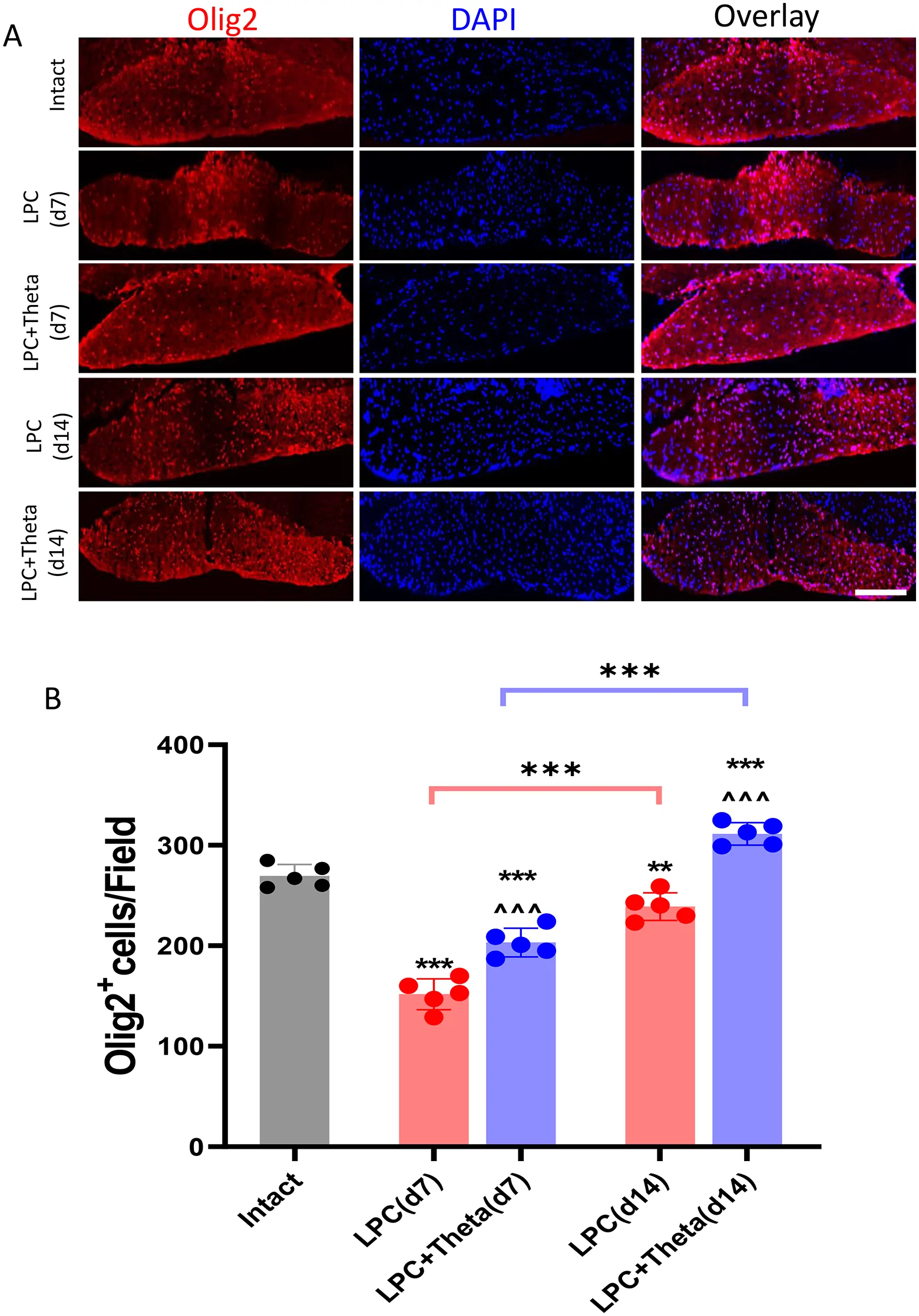
OLIG2+ cell numbers after LPC-induced demyelination and theta entrainment. (A) Representative immunofluorescence images showing OLIG2 immunoreactivity (green) and DAPI nuclear staining (blue) in the Intact, LPC day 7, LPC+Theta day 7, LPC day 14, and LPC+Theta day 14 groups. (B) Quantification of OLIG2+ cells per field in the analyzed optic chiasm region. Data are presented as mean ± SEM. Statistical analysis was performed using two-way ANOVA followed by Tukey’s multiple-comparisons test. ***p < 0.001 versus Intact; ^^p < 0.01, ^^^p < 0.001 versus the corresponding LPC group.

**Fig 10:**
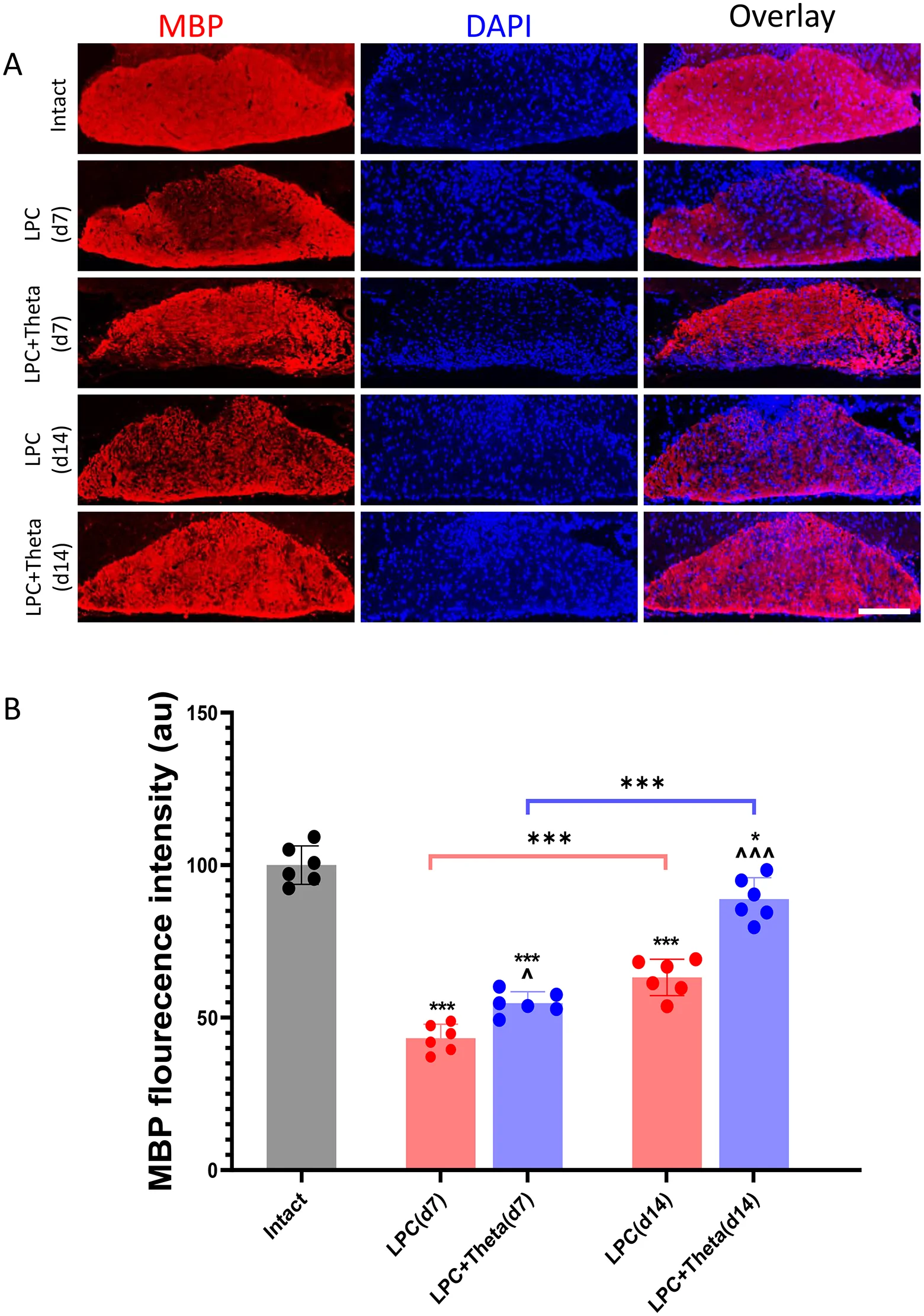
MBP immunoreactivity after LPC-induced demyelination and theta entrainment. (A) Representative immunofluorescence images showing MBP immunoreactivity (red) and DAPI nuclear staining (blue) in the Intact, LPC day 7, LPC+Theta day 7, LPC day 14, and LPC+Theta day 14 groups. (B) Quantification of MBP fluorescence intensity normalized to the Intact group. Data are presented as mean ± SEM. Statistical analysis was performed using two-way ANOVA followed by Tukey’s multiple-comparisons test. *p < 0.05, **p < 0.01, ***p < 0.001 versus Intact; ^^p < 0.01, ^^^p < 0.001 versus the corresponding LPC group.

To evaluate protein expression following LPC induced demyelination with and without theta stimulation, immunofluorescence staining for PDGFRα, OLIG2, and MBP was performed and fluorescence intensity was quantified (Figs 8–10). PDGFRα expression was significantly upregulated in LPC treated groups at both day 7 and day 14 post injection compared with the intact group (Fig 8A). At day 7, LPC injection led to a marked increase in PDGFRα levels (***P < 0.001 vs. intact). The LPC + Theta group showed a further increase in PDGFRα expression (***P < 0.001 compare to intact and ^^^P < 0.001 compare to LPC(d7)). By day 14, PDGFRα expression remained elevated in the LPC group (***p < 0.001 vs. intact). However, in the LPC + Theta group, PDGFRα expression was significantly reduced compared to the LPC only group at the same time point (^^^p < 0.001) (Fig 8B). Two‑way ANOVA revealed significant effects of experimental group (intact, LPC, and LPC+Theta; F (2,24) =207.4, p < 0.0001), time (days 7 and 14; F(1,24)=15.00, p = 0.0007), and their interaction (F(2,24)=93.68, p < 0.0001) on PDGFRα expression.

OLIG2 immunofluorescence staining was performed to assess oligodendrocyte lineage cells (Fig 9). In the intact optic chiasm, a high baseline number of OLIG2+ cells was observed (Fig 9A). At day 7 post LPC injection, OLIG2+ cells significantly decreased in the demyelinated region (p < 0.001 vs. intact) (Fig 9B). Theta stimulation at day 7 (LPC + Theta Day 7) resulted in a statistically significant increase in OLIG2+ cells compared to LPC (^^^p < 0.001). Although OLIG2+ cell counts did not reach intact levels, they were significantly higher than LPC day 7. By day 14, the number of OLIG2+ cells increased in the LPC group compared to day 7 (**p < 0.01 vs. LPC day 7). When theta stimulation was applied (LPC + Theta (d14)), a significant enhancement in Olig2+ cells was observed compared to both LPC day 14 and LPC + Theta Day 7 groups (***p < 0.001). Two‑way ANOVA demonstrated significant effects of experimental group (intact, LPC, and LPC+Theta; F(2,24)=116.8, p < 0.0001), time (days 7 and 14; F(1,24)=235.0, p < 0.0001), and their interaction (F(2,24)=60.57, p < 0.0001) on OLIG2+ cell counts. MBP expression was evaluated at days 7 and 14 (Fig 10A). In intact tissue, MBP expression was robust. Following LPC injection at day 7, MBP fluorescence intensity significantly decreased to approximately 50% of intact levels (p < 0.001). In the LPC + Theta (d7) group, MBP expression remained around 50% of intact levels and did not show a significant increase compared with LPC alone (^p < 0.05 compare to LPC (d7)). By day 14, the LPC group exhibited partial recovery of MBP expression, although levels remained significantly lower than intact (p < 0.001). In contrast, the LPC + Theta Day 14 group showed a further significant increase in MBP fluorescence intensity (^^^p < 0.001 compared to LPC at day 14) (Fig 10B). Two‑way ANOVA revealed significant effects of experimental group (intact, LPC, and LPC+Theta; F(2,45)=351.0, p < 0.0001), time (days 7 and 14; F(2,45)=52.10, p < 0.0001), and their interaction (F(4,45)=17.17, p < 0.0001) on MBP fluorescence intensity. Together, these immunofluorescence findings suggest that theta entrainment was associated with changes in oligodendrocyte-lineage and myelin-associated protein markers after LPC-induced demyelination.

## 4 Discussion

The results of this study indicate that non-invasive theta oscillation entrainment (4 Hz) was associated with changes in VEP latency and with molecular and cellular changes related to myelin-associated responses following LPC-induced demyelination in the optic chiasm. The observed reduction in VEP latency at days 7 and 14, in conjunction with alterations in oligodendrocyte lineage markers and inflammatory profiles, suggests that patterned neuronal activity in the theta range may influence the microenvironment of a demyelinated lesion in ways associated with recovery. These findings provide experimental support for the potential involvement of activity-dependent mechanisms in myelin plasticity, while highlighting the need for further studies to determine the underlying mechanisms and to assess whether these effects extend to chronic or immune-mediated models of demyelination.

One notable observation was the reduction in P1 wave latency in Visual Evoked Potentials (VEPs) across the examined time points, reaching significance by day 14. The observed reduction in P1 latency may reflect changes in visual pathway conduction following demyelination; however, a direct causal relationship cannot be established from the present data [33,34]. These observations are consistent with previous reports suggesting that synchronized neuronal activity may influence neuroplasticity following injury [34], including processes potentially related to myelin repair [35,36]. At the molecular and cellular levels, we observed reductions in *Aif-1* and Gfap expression, confirmed by immunohistochemistry and gene expression analysis. Because glial activation can influence the microenvironment within demyelinated lesions, these changes may reflect altered inflammatory responses during recovery [37,38]. However, *Aif-1* and Gfap alone are not sufficient to definitively define microglial activation or astrocyte reactivity. A more precise characterization of microglial activation would require additional activation-associated markers, such as CD68 or MHC-II, together with morphological analysis or functional assays [39]. Similarly, defining astrocyte reactivity would require complementary GFAP immunostaining, morphological assessment, and preferably additional astrocyte markers such as ALDH1L1 or S100β [40].Previous studies have suggested that neural oscillations may interact with neuroimmune signaling pathways [41–43], although the specific mechanisms underlying these observations remain to be determined [44–46].

In addition, increased Myelin Basic Protein (MBP) levels were observed by day 14, which may reflect partial restoration of myelin‑related protein expression during the recovery phase [47]. Importantly, the present experimental design does not allow a clear distinction between reduced initial demyelination (i.e., neuroprotection or myelin preservation) and accelerated remyelination. Because differences between the LPC and LPC+Theta groups were already detectable at earlier time points, the increased expression of myelin related markers observed at later stages may reflect either reduced early myelin damage or enhanced recovery processes. Distinguishing between these possibilities would require dedicated temporal analyses or lineage‑tracing approaches that directly track newly generated oligodendrocytes and myelin formation, as discussed in studies of remyelination dynamics in the CNS [48].

At the oligodendrocyte-lineage level, theta entrainment was associated with changes in *Olig2*, *Pdgfra*, and *Plp1* transcript profiles following LPC-induced demyelination. In the LPC group, reduced Olig2 transcript levels and decreased OLIG2+ cell numbers at day 7 were consistent with oligodendrocyte-lineage disruption during the peak demyelination phase. In contrast, the LPC+Theta group showed relatively higher Olig2 transcript levels and OLIG2+ cell numbers compared with the corresponding LPC group, suggesting modulation of oligodendrocyte-lineage-associated responses. In addition, higher *Pdgfra* transcript levels at early time points and increased *Plp1* expression by day 14 in the theta-treated group may reflect changes in OPC-associated and myelin-associated responses during the lesion-recovery period. However, because *Olig2* is broadly expressed across the oligodendrocyte lineage, these findings should be interpreted as reflecting changes in the abundance or state of oligodendrocyte-lineage cells rather than direct evidence of OPC differentiation. Similarly, although *Pdgfra* expression is associated with OPCs, *Pdgfra* alone cannot distinguish between increased OPC proliferation, recruitment, retention, or survival. Therefore, the present data indicate changes in oligodendrocyte-lineage-associated markers rather than definitive evidence of lineage progression or accelerated differentiation. Previous studies have proposed that neuronal activity may influence myelination processes (25, 48). However, determining whether theta entrainment directly regulates OPC proliferation or oligodendrocyte differentiation will require targeted mechanistic studies using proliferation markers such as Ki67, EdU, or BrdU, together with stage-specific lineage markers such as PDGFRα/NG2 and mature oligodendrocyte markers, or lineage-tracing approaches (45, 47, 49, 50).

However, before broader conclusions regarding therapeutic applications can be drawn, key questions remain. Future research must determine the optimal stimulation parameters (frequency, duration, and intensity) [49,50]. Furthermore, verifying whether similar effects occur in other CNS regions and exploring its potential interaction with pharmacological agents that promote differentiation [51–54] represent important directions for future investigation. Although this study was conducted in a murine model of LPC-induced demyelination, caution should be exercised when extrapolating these findings to other species or clinical conditions. Further studies in additional models and experimental systems will be necessary to evaluate potential translational relevance. This study adhered to the principles of the 3Rs. The number of animals was minimized through careful experimental design and the use of multiple outcome measures per animal (reduction). Non-invasive visual stimulation was employed to refine experimental procedures and minimize animal distress (refinement). Several limitations should be acknowledged. The sample size was relatively small, which may limit statistical power. In addition, only male mice were used, and findings may not be generalizable to females. The LPC-induced demyelination model represents an acute injury model and may not fully recapitulate the chronic and immune-mediated aspects of human demyelinating diseases.

A key limitation of the present study is that the theta-stimulated group already exhibited reduced demyelination at earlier time points. As a result, the current design cannot clearly determine whether the effects observed at later stages reflect reduced initial myelin damage (neuroprotection/myelin preservation) or a true acceleration of remyelination. Future studies incorporating experimental designs that temporally separate these processes will be necessary to address this distinction.

In summary, the results indicate that theta oscillation entrainment was associated with changes in VEP latency and in inflammatory and oligodendrocyte lineage markers following LPC-induced demyelination in the optic chiasm. These observations suggest that patterned neuronal activity may influence processes related to myelin repair. Further mechanistic, behavioral, and translational studies are required to determine causality and to assess the broader relevance of theta entrainment in demyelinating conditions.

## Supporting information

S1 Table Summary of experimental analyses and statistical methods. (XLSX)

S1 Data Raw data underlying Figures 1–10. (XLSX)
